# The effect of Toll-like receptor agonists on the immunogenicity of MVA-SARS-2-S vaccine after intranasal administration in mice

**DOI:** 10.3389/fcimb.2023.1259822

**Published:** 2023-10-03

**Authors:** Kim Thi Hoang Do, Stefanie Willenzon, Jasmin Ristenpart, Anika Janssen, Asisa Volz, Gerd Sutter, Reinhold Förster, Berislav Bošnjak

**Affiliations:** ^1^ Institute of Immunology, Hannover Medical School, Hannover, Germany; ^2^ Institute for Virology, University of Veterinary Medicine Hannover, Hannover, Germany; ^3^ German Centre for Infection Research (DZIF), Munich, Germany; ^4^ Division of Virology, Department of Veterinary Sciences, Ludwig Maximiliam University (LMU) Munich, Munich, Germany; ^5^ Cluster of Excellence RESIST (EXC 2155), Hannover Medical School, Hannover, Germany; ^6^ German Centre for Infection Research (DZIF), Hannover, Germany

**Keywords:** modified vaccinia virus Ankara (MVA), respiratory tract, severe acute respiratory syndrome coronavirus 2 (SARS-CoV-2), Toll-like receptor (TLR) agonist, vaccination

## Abstract

**Background and aims:**

Modified Vaccinia virus Ankara (MVA) represents a promising vaccine vector for respiratory administration to induce protective lung immunity including tertiary lymphoid structure, the bronchus-associated lymphoid tissue (BALT). However, MVA expressing the severe acute respiratory syndrome coronavirus 2 (SARS-CoV-2) Spike protein (MVA-SARS-2-S) required prime-boost administration to induce high titers of anti-Spike antibodies in serum and bronchoalveolar lavage (BAL). As the addition of adjuvants enables efficient tailoring of the immune responses even to live vaccines, we tested whether Toll-like receptor (TLR)-agonists affect immune responses induced by a single dose of intranasally applied MVA-SARS-2-S.

**Methods:**

We intranasally immunized C57BL/6 mice with MVA-SARS-2-S vaccine in the presence of either TLR3 agonist polyinosinic polycytidylic acid [poly(I:C)], TLR4 agonist bacterial lipopolysaccharide (LPS) from *Escherichia coli*, or TLR9 agonist CpG oligodeoxynucleotide (CpG ODN) 1826. At different time-points after immunization, we analyzed induced immune responses using flow cytometry, immunofluorescent microscopy, and ELISA.

**Results:**

TLR agonists had profound effects on MVA-SARS-2-S-induced immune responses. At day 1 post intranasal application, the TLR4 agonist significantly affected MVA-induced activation of dendritic cells (DCs) within the draining bronchial lymph nodes, increasing the ratio of CD11b^+^CD86^+^ to CD103^+^CD86^+^ DCs. Nevertheless, the number of Spike-specific CD8^+^ T cells within the lungs at day 12 after vaccination was increased in mice that received MVA-SARS-2-S co-administered with TLR3 but not TLR4 agonists. TLR9 agonist did neither significantly affect MVA-induced DC activation nor the induction of Spike-specific CD8^+^ T cells but reduced both number and size of bronchus-associated lymphoid tissue. Surprisingly, the addition of all TLR agonists failed to boost the levels of Spike-specific antibodies in serum and bronchoalveolar lavage.

**Conclusions:**

Our study indicates a potential role of TLR-agonists as a tool to modulate immune responses to live vector vaccines. Particularly TLR3 agonists hold a promise to potentiate MVA-induced cellular immune responses. On the other hand, additional research is necessary to identify optimal combinations of agonists that could enhance MVA-induced humoral responses.

## Introduction

The recent coronavirus disease 2019 (COVID-19) pandemic highlighted the importance of vaccination as an effective measure against infectious diseases. Licensed COVID-19 vaccines successfully prevent severe infection and death from COVID-19 but provide significantly less protection against mild or asymptomatic disease (Yang et al., 2023). This incomplete protection is partially attributable to the emergence and prevalence of novel virus strains that accumulated non-synonymous mutations affecting the function of crucial viral proteins, including the Spike (Bansal and Kumar, 2022). Mutations within the Spike protein of Omicron B.1.1.529/BA sublineages such as BA.1, BA.2, and BA.5, resulted in a significant reduction in sensitivity to neutralizing antibodies [reviewed in (Carabelli et al., 2023)]. At the same, these mutations also modified virus entry mechanisms, thus reducing the latency time required for mobilization of systemic memory B and T cells induced by vaccination (Carabelli et al., 2023). Another important factor for incomplete protection is the lack of effective protective immunity within the respiratory mucosa. Systemically induced immunoglobulin (Ig) G (IgG) antibodies can passively leak into and protect respiratory passages only if they are present in high titers in serum (Lund and Randall, 2021). Moreover, effective protection of mucosal surfaces requires the presence of protective tissue-resident memory B and T cells, which cannot be induced without antigen delivery directly to the respiratory tract (McMaster et al., 2018; Allie et al., 2019).

To efficiently induce anti-viral immune responses at the point of SARS-CoV-2 entry, others and we have suggested respiratory delivery of COVID-19 vaccines (Förster et al., 2020; Lund and Randall, 2021). In preclinical models, respiratory delivery of the COVID-19 vaccines generates, in addition to systemic responses, also neutralizing IgA antibodies within the bronchoalveolar lavage (Feng et al., 2020; Bošnjak et al., 2021; Afkhami et al., 2022). Mucosal IgA has the potential to neutralize the SARS-CoV-2 before it can penetrate the body (Lamers and Haagmans, 2022). If the case that SARS-CoV-2 breaches the IgA-mediated protection, vaccine-induced tissue-resident SARS-CoV-2 specific T cells provide a second layer of defense, either directly killing infected cells or recruiting other leukocytes (Feng et al., 2020; Bošnjak et al., 2021; Afkhami et al., 2022). Together, these mucosal anti-SARS-CoV-2-specific responses efficiently protect experimental animals from SARS-CoV-2 infection (Feng et al., 2020; Bošnjak et al., 2021). More importantly, preclinical data indicate that mucosal immune responses induced by respiratory-delivered COVID-19 vaccines not only efficiently clear SARS-CoV-2 infection (An et al., 2021; Bošnjak et al., 2021; Bricker et al., 2021; Ku et al., 2021) but also reduce viral replication and shedding from the respiratory tract, thus preventing SARS-CoV-2 transmission (Van Doremalen et al., 2021; Langel et al., 2022).

A respiratory vaccination would be, therefore, a preferable response to a potential natural outbreak of novel respiratory pathogens. Ideally, these vaccines would be administered as a single dose, thus enabling almost immediate protection in contrast to vaccines requiring multiple dosages over an extended period. A particularly interesting respiratory vaccine vector is MVA, a highly attenuated vaccinia virus already licensed as smallpox and monkeypox vaccine in Canada, European Union, and the United States (Volz and Sutter, 2017). We have previously shown that respiratory delivery of an MVA-based COVID-19 vaccine candidate, MVA-SARS-2-S, in prime-boost protocols induces potent protective humoral and cellular immune responses directed against the SARS-CoV-2 Spike protein in rodent animal models (Bošnjak et al., 2021). Already a single MVA-SARS-2-S intranasal dose induced the generation of potent systemic and respiratory T cell responses, anti-Spike specific antibodies in serum, and bronchus-associated lymphoid tissue (BALT) (Bošnjak et al., 2021), a tertiary lymphoid structure that supports the activation of T and B cells specific to pathogens that penetrate the lower respiratory tract (Halle et al., 2009; Tan et al., 2019). However, the induction of protective neutralizing IgA responses directed against the Spike protein within the respiratory tract required vaccine administration in prime-boost protocols (Bošnjak et al., 2021).

Immunization properties of viral vector vaccines primarily depend on their intrinsic ability to induce both humoral and cellular adaptive immune responses. In the case of MVA, its immunization properties primarily depend on the activation of cyclic GMP–AMP synthase (cGAS)–stimulator of interferon genes (STING) pathway in dendritic cells (Waibler et al., 2007; Liu et al., 2008; Dai et al., 2014; Dai et al., 2017; Döring et al., 2021). Ample evidence indicates that the immunogenicity of viral vector vaccines, including MVA, can be further enhanced or modulated by adjuvants (Belyakov et al., 2006; Israely et al., 2014; Magnusson et al., 2018; Sanos et al., 2018; Mahony, 2021; Manohar et al., 2022). Some of the main adjuvant targets are TLRs, whose activation potentiates innate and adaptive immune responses (Sartorius et al., 2021; Kaur et al., 2022). Hence, TLR-based immune adjuvants could be used to boost MVA-induced immune responses. However, activation of different pattern recognition receptors can also lead to antagonistic rather than synergistic effects on the immune response (Lee and Kim, 2007). Moreover, immune-stimulatory properties of TLR agonists can be used to combat viral infections [reviewed in (Owen et al., 2021)], further suggesting that they could have antagonizing effects on the vaccination with viral vectors. Therefore, we aimed to determine which TLR would represent the prime target to boost immune responses to the respiratory delivered MVA-SARS-2-S vaccine. We found that the addition of different TLR agonists affected distinct aspects of the immune response induced by the vaccine. Importantly, the TLR3 agonist poly(I:C) potentiated the generation of vaccine-induced S-specific CD8^+^ T cells. The addition of the TLR4 agonist LPS did not affect cellular immunity, despite increasing the migration of CD11b^+^CD86^+^ DC from the lungs into the bronchial lymph nodes (bLNs). Interestingly, the TLR9 agonist CpG oligonucleotide (ODN) inhibited the development of BALT. On the other hand, all the investigated TLR agonists suppressed the levels of S-specific antibodies in serum and bronchoalveolar lavage (BAL), highlighting the complexity of TLR signaling in the regulation of B cell responses. Together, these results provide important considerations for the selection of TLRs as adjuvant targets for viral vector vaccines.

## Materials and methods

### Experimental animals

C57BL/6N mice were purchased from Charles River and maintained at the Central Animal Facility of Hanover Medical School (Hanover, Germany) under specific pathogen-free conditions with free access to food and water. They were used for experiments at the age of 12-16 weeks, with a minimum of a one-week adaptation period to the experimental animal room.

### MVA-SARS-2-S immunization

The recombinant MVA-SARS-2-S was described previously (Tscherne et al., 2021). For intranasal immunization, mice were anesthetized by intraperitoneal administration of a combination of 50 mg/kg ketamine and 10 mg/kg xylazine. Subsequently, the mice were immunized with 10^7^ plaque-forming units (PFU) of MVA-SARS-2-S resuspended in total volume of 40 μl saline containing 15 mM Tris pH 7.7, 3% sucrose, and 0.005% Tween 80. In the groups receiving MVA with TLR agonists, MVA was administered together with 5 µg LPS from *Escherichia coli* O55:B5 (Cat.# L2637, Sigma-Aldrich), 5 µg class B CpG ODN 1826 (Cat.# tlrl-1826, InvivoGen), or 3 µg poly(I: C) (Cat.# P1530, Sigma-Aldrich).

### Sample collection and preparation of single cell suspension

We collected the samples at early (22 hours and 48 hours) and late (day 12) time points after immunization (p.i.). To label leukocytes within the blood vessels, we intravenously administered 5 µg of FITC-CD45 (Clone 30-F11; **Supplementary Table S1**) into mice terminally anesthetized by gradual exposure to carbon dioxide (CO_2_). Within 5 minutes after antibody injection, saphenous vein blood was collected, allowed to clot, and centrifuged to separate the serum as supernatant, which was stored at -20°C until further analyses.

Following the blood collection procedure, the spleens were resected. Subsequently, broncho-alveolar lavage (BAL) was collected and separated using centrifugation into BAL fluid and cells as described earlier (Bošnjak et al., 2021). After BAL collection, all organs were excised from the chest cavity. First, the bronchial lymph nodes (bLNs) and right lung lobes separated for immune cell phenotyping as described below. Then the left lung lobe was inflated through the bronchus with a mixture of optimal cutting temperature (OCT): PBS (1:1) and frozen in OCT (Tissue-Tek) on dry ice for histological analysis.

Right lung lobes were next inflated with #1 digestion media made of 4 U/ml of Elastase (cat.# LS002292, Worthington Biochemical Corporation), 1 U/ml of Dispase II (cat.# 4942078001, Sigma-Aldrich), 200 µg/ml of DNase I (cat.# 11284932001, Sigma-Aldrich) diluted in RPMI without phenol red (cat.# 11-835-055, Gibco). Inflated lung lobes were then incubated in a well-containing 2 ml of #1 digestion media for 30 mins at 37°C. Afterward, lung lobes were minced into small pieces, which were then transferred into a 50-mL conical tube containing 5 ml of digestion medium #2 (RPMI without phenol red containing 5% of FCS, 25 µg/ml of Liberase (cat.# 5401127001, Roche), and 200 µg/ml of DNase I (Sigma-Aldrich). After the second incubation for 30 minutes at 37°C, single lung cells were separated by passing the samples through 40 μm cell strainers. The red blood cells (RBCs) were removed using 1X erythrocyte lysis buffer and samples were filtered again through a 40 μm cell strainer before immunophenotyping using flow cytometry.

The bLN capsule was pierced using forceps to facilitate the digestion process. Next, the bLNs were transferred into Eppendorf tubes containing 1 ml of DB digestion media consisting of RPMI without phenol red (Gibco), 90 μg/ml Liberase (Roche), and 10 μg/ml DNase I (Sigma-Aldrich). The samples were shaken at 300 rpm at 37°C for 20 minutes in a Thermomixer. Afterward, the samples were pipetted using a 1 ml pipette up and down ten times to dissolve any remaining tissues. After undigested parts settled on the bottom of the tube, the supernatant was transferred into a 15 ml tube filled with MACS buffer (PBS containing 3% FBS and 5 mM EDTA). The fresh DB media was added to the undigested bLN parts, and the process was repeated for two additional rounds to completely dissolve all remaining tissue. Finally, erythrocytes were removed by hypertonic red blood cell lysis and samples were filtered through 40 μm cell strainer.

Spleens were obtained by triturating the organs through 40 μm cell strainers. Erythrocyte lysis buffer was applied to eliminate red blood cells and the splenocytes were then filtered through 40 μm cell strainers before analysis using flow cytometry.

### Leukocyte immunophenotyping using spectral flow cytometry

To minimize the nonspecific antibody binding, the samples were initially incubated with 10% rat serum for 10 minutes at 4°C. After blocking, antibody mixes (**Supplementary Table S1; Panel 1**) were added to the samples collected at 22-48 hours p.i. without washing. After additional 30 minutes of incubation at 37°C, the samples underwent two times of washing with MACS buffer to remove any unbound antibodies before proceeding with flow cytometry analysis. For the analysis of samples collected at day 12 p.i., the cells were after blocking first incubated with tetramers loaded with MVA and SARS-CoV-2-S peptides for 15 minutes at 37°C. The tetramers were prepared by conjugating Vaccinia virus WR peptide B8R 20-27 (TSYKFESV) or an 8-amino acid immunodominant spike peptide V8L 539-546 (VNFNFNGL; both peptides from GenScript) onto empty H-2kb tetramers according to the manufacturer’s protocol (Tetramer-Shop). Next, the antibody mixes (**Supplementary Table S1; Panel 2**) were added without washing and the samples were further incubated for 15 minutes at 37°C. After appropriate washing, the samples were acquired using a Cytek Aurora spectral flow cytometer (Cytek) equipped with lasers operating at 355nm, 405nm, 488nm, 561nm, and 640nm. The acquired flow cytometry data were subsequently analyzed using FCS Express V7 (Denovo) or FlowJo V10 (BD) software. For visualization, we used UMAP pipeline of the FCS Express. For quantification, we used the classical gating strategies depicted in the **Supplement Figures**. The percentage of each cell type of interest was expressed as a frequency of total live cells. Then, these frequencies were multiplied by the total number of cells isolated from the organ to obtain the absolute number of cells within the population.

### Immunohistology

Frozen mouse’s lung tissue blocks were sliced into 8 µm thick sections and subsequently fixed in acetone for 15 minutes on ice. For analysis, the cryosections containing the primary bronchi were rehydrated in TBS (PBS with 0.05% Tween) for 5-10 minutes and then washed two times using TBS. Next, non-specific antibody binding was blocked by incubation with a mixture of 5% rat serum and 5% anti-CD16/CD32 antibody (clone 2.4G2, produced in-house) in TBS for 15 minutes. Subsequently, the cryosections were incubated with the antibodies mixed (see **Supplementary Table S1**) for 45 minutes at room temperature, followed by two TBS washes. DAPI staining (1 µg/ml) was performed for 3 minutes, and another round of TBS wash was carried out before embedding the sections in the FluorSaveTM reagent (cat.# 345789, Millipore). The sections were imaged using an Axioscan Z1 microscope (Zeiss). The quantification of bronchus-associated lymphoid tissue (BALT) was conducted using ZEN 3.2 blue software (Zeiss) as described previously (Fleige and Förster, 2017). Briefly, panoramic images covering the entire sections from various central planes (in proximity to the main bronchi and vessels) were captured and analyzed. Individual BALT structures were enumerated, and their surface area was measured. Finally, the cumulative BALT size was calculated by summing the surface areas of all individual BALT structures present in a single central lung section.

### Enzyme-linked immunosorbent assay

We determined the titer of anti-spike IgG antibodies in sera or BAL samples using ELISA as described in detail earlier (Bošnjak et al., 2021). Briefly, serial dilutions of samples were incubated on ELISA plates coated with SARS-CoV-2-S trimer fused to mNEONgreen and blocked with 2% bovine serum albumin (BSA). Afterwards, anti-spike specific isotype antibodies were detected using goat anti-mouse IgG-Fc antibody HRP-conjugated (cat.# 1013-05, SouthernBiotech) and TMB Substrate Reagent (cat.# 555214, BD Biosciences), and quantified in duplicates using a SpectraMax iD3 microplate reader (Molecular Devices).

### Statistical analysis

To analyze statistical differences between the groups, we used the Brown-Forsythe ANOVA test followed by Dunnett’s T3 multiple comparisons test or Two-way ANOVA. The numbers of different cell populations were log-transformed before statistical comparison to equalize the standard deviation between the groups.

## Results

### The addition of TLR-agonists does not exaggerate early immune response to intranasally administered MVA-SARS-2-S

**Figure 1:**
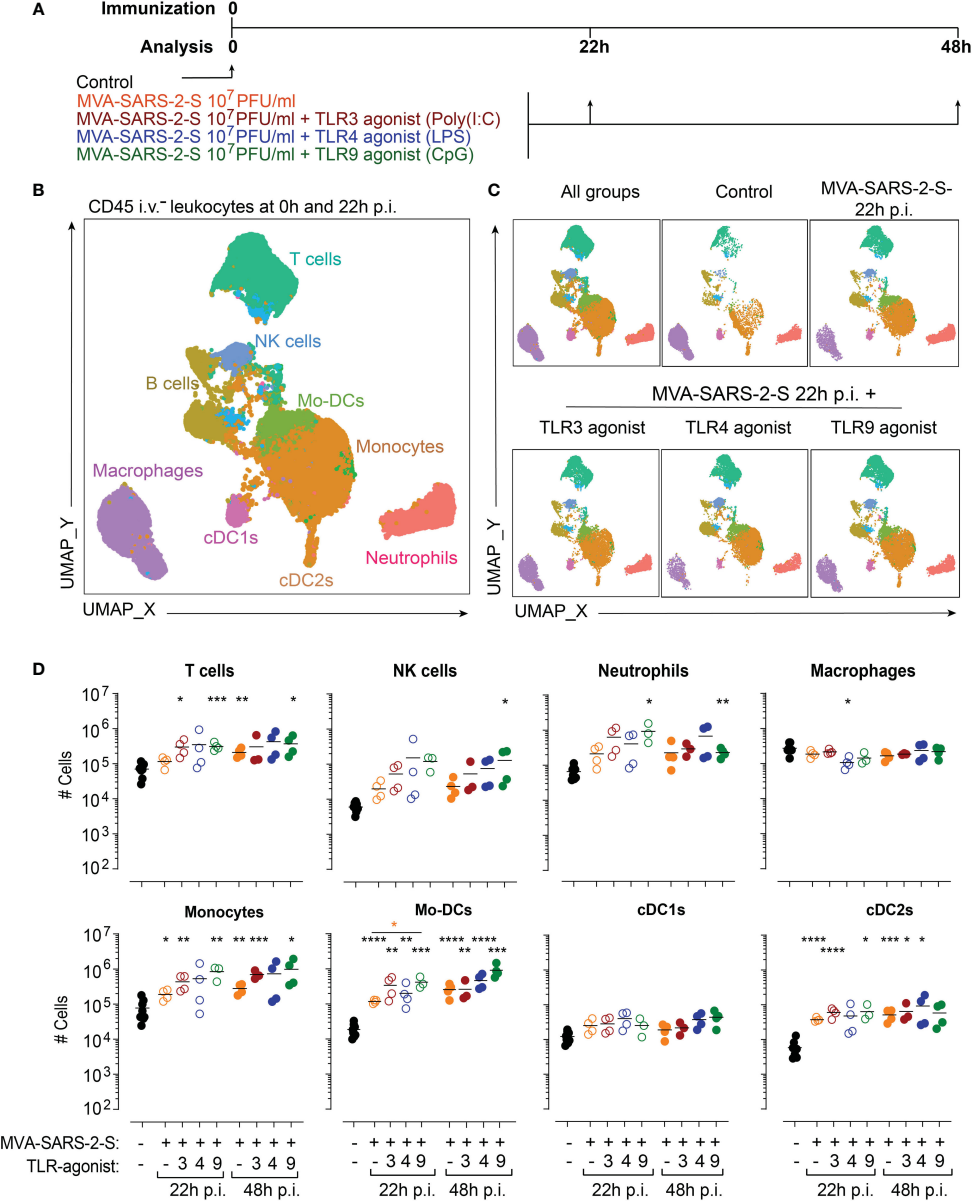
TLR agonists do not exaggerate MVA-SARS-2-S induced acute inflammation in the lungs. **(A)** Scheme of immunization protocol. **(B, C)** Leukocyte composition within lung parenchyma presented as a UMAP plot generated from concatenated samples of two representative mice from each experimental group at 24 hours p.i. (two independent experiments with n = 3-4 mice per group). Spectral flow cytometry data are gated as depicted in **Supplementary Figure 1A** using antibodies listed in **Supplementary Table S1** (Panel 1). **(D)** Absolute cell numbers of T cells, NK cells, neutrophils, macrophages, monocytes, monocyte-derived dendritic cells (Mo-DCs), conventional dendritic cells type 1 (cDC1s) and type 2 (cDC2s). Individual values (symbols) and mean group value (lines) pooled from two experiments with n = 3-4 per group. Statistical analysis was done on log-transformed using Brown-Forsythe ANOVA test followed by Dunnett’s T3 multiple comparisons test. *p < 0.05, **p < 0.01, ***p < 0.001, ****p < 0.0001. Black stars - difference to control group; Orange stars – difference between groups receiving vaccine and vaccine mixed with TLR agonists, as denoted by the line.

TLRs have broad and distinct expression patterns on lung immune and structural cells (Arora et al., 2019). Hence, intranasal administration of poly(I:C) targeting TLR3, LPS targeting TLR4, and CpG ODN 1826 targeting TLR9 induces varying inflammatory cascades leading to acute lung inflammation (Bosnar et al., 2009; Harris et al., 2013; Kim et al., 2019). To gain insight into how co-administration of each investigated TLR agonist affects the MVA-SARS-2-S-induced innate immune response, we analyzed leukocyte cell subsets in mouse lungs during 48hr post vaccine administration (**Figure 1A**). For vaccination, we have chosen 10^7^ PFU of MVA-SARS-2-S, as lower MVA-SARS-2-S doses failed to induce protective neutralizing responses directed against the Spike protein in BAL (data not shown). The doses of TLR agonists were selected according to literature data confirming their adjuvant effects (Ichinohe et al., 2005; Bhat et al., 2009; Rhee et al., 2010; Uppada et al., 2011; Lebedeva et al., 2018).

To visualize differences in lung cell populations, we used unsupervised clustering of leukocytes within the lung parenchyma stained with antibody panel 1 and analyzed using spectral flow cytometry. Resulting Uniform Manifold Approximation and Projection (UMAP) plots revealed profound changes in immune cell composition 22 hours after intranasal immunization (**Figures 1B, C**). Next, we quantified in detail the infiltrating leukocyte populations (see material and methods and **Supplementary Figure 1A**). We found that vaccination with MVA-SARS-2-S was predominantly characterized by a significant accumulation of myeloid cells, including monocyte-derived DCs (Mo-DCs; 38.5x and 125x higher mean number at 22 and 48 hours, respectively), conventional dendritic cells type 2 (cDC2s; 6.3x and 8.5x higher mean number at 22 and 48 hours, respectively), and monocytes (2.4x and 3.6x higher mean number at 22 and 48 hours, respectively), while the numbers of conventional dendritic cells type 1 (cDC1s) did not change significantly (2.0x and 1.5x higher mean number at 22 and 48 hours, respectively; **Figure 1D**). Additionally, the numbers of NK cells and neutrophils also increased, although not significantly, while the numbers of T cells, B cells, and macrophages did not change (**Figure 1D**; **Supplementary Figure 1B**). Importantly, TLR3 or TLR4 agonists had only minimal effects on vaccine-induced immune response (**Figure 1D**; **Supplementary Figure 1B**). The addition of TLR9 agonist, on the other hand, selectively increased the Mo-DC number and did not affect the accumulation of other cell populations (**Figure 1D**; **Supplementary Figure 1B**). Together, these data suggest that the co-administration of TLR agonists does not exaggerate immune response initiated by respiratory delivery of MVA-SARS-2-S.

### TLR4 agonist increased the proportion of MVA-SARS-2-S-activated cDC2 in bLNs

**Figure 2:**
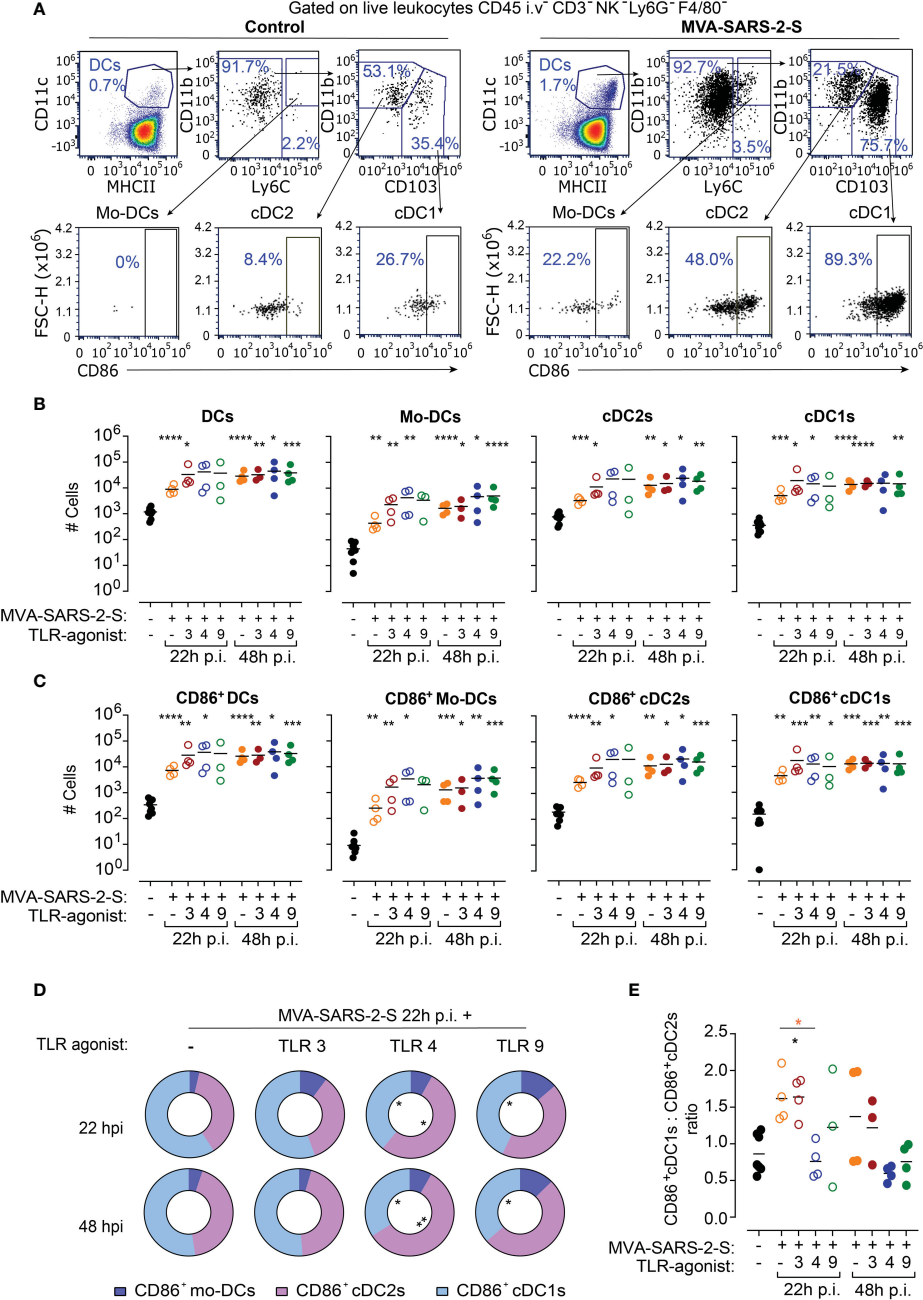
TLR4 agonists increased proportion of cDC2 cells in bronchial lymph nodes (bLNs) at 24 hours (h) after MVA-SARS-2-S immunization. **(A)** Representative dot plots identifying absolute and activated (CD86^+^) DCs and their subpopulations: monocyte-derived dendritic cells (Mo-DCs), conventional dendritic cells type 1 (cDC1s) and type 2 (cDC2s) in bLNs of indicated groups. **(B, C)** Absolute cell numbers **(B)** and number of CD86+ **(C)** total DCs, Mo-DCs, cDC2s, and cDC1s. **(D)** Relative distribution of CD86^+^Mo-DCs, CD86^+^cDC1s, and CD86^+^cDC2s in indicated groups. **(E)** Ratio between CD86^+^cDC1s and CD86^+^cDC2s across different analyzed groups. **(B–E)** Pooled data from two experiments with n=3-4 mice per group. **(B, C, E)** Individual values (symbols) and mean group value (lines). Statistical analysis was done on log-transformed **(B, C)** or ratios **(E)** using Brown-Forsythe ANOVA test followed by Dunnett’s T3 multiple comparisons test and Two-way ANOVA **(D)**. *p < 0.05, **p < 0.01, ***p < 0.001, ****p < 0.0001. Black stars - difference to control group; Orange stars – difference between groups receiving vaccine and vaccine mixed with TLR agonists, as denoted by the line.

For the successful initiation of T cell responses to vaccination, DCs have to capture antigens, get activated, and migrate to the draining lymph nodes. Hence, we next determined how the addition of TLR agonists affected DC migration from the lungs into the bLNs at 22 and 48 hours post-vaccination using spectral flow cytometry (**Figure 2A**; **Supplementary Figure 2**). In line with our previous results (Halle et al., 2009), MVA-SARS-2-S vaccination induced a rapid increase in the absolute numbers of DCs in bLNs (21x and 75x higher mean number at 22 and 48 hours, respectively; **Figure 2B**). Detailed characterization indicated an increase in the number of all three investigated DC subtypes, cDC1s, cDC2s, and MO-DCs, in the MVA-SARS-2-S vaccinated group compared to the control mouse group (**Figure 2B**). Importantly, all bLN DC subtypes of i.n. MVA-SARS-2-S vaccinated mice expressed higher levels of the co-stimulatory molecule CD86 compared to non-vaccinated mice (**Figure 2C**), suggesting that they could productively initiate an adaptive immune response to the vaccine.

Interestingly, the addition of TLR agonists to the vaccine did neither significantly affect numbers nor activation of bLN DCs in comparison with the group treated only with MVA-SARS-2-S (**Figures 2B, C**). However, a detailed analysis indicated that the TLR4 agonist affected the proportions of the activated DC subsets in the bLNs (**Figures 2D, E**). In bLNs of mice vaccinated with MVA-SARS-2-S, cDC1 was the most abundant CD86^+^ DC subtype (59.2% ± 6.2% and 52.4% ± 14.3% at 22 and 48 hours, respectively). The addition of TLR3 or TLR9 agonists to the vaccine did not significantly affect the MVA-SARS-2-S-induced DC activation in bLNs. On the other hand, CD86^+^cDC2s were the largest activated DC subpopulation (52.9% ± 6.2% and 57.8% ± 4.1% at 22 and 48 hours, respectively) in bLNs of mice that received the vaccine combined with TLR4 agonist, resulting in a change of the ratio between CD103^+^CD86^+^ to CD11b^+^CD86^+^ DCs at 22 hours after vaccine administration (**Figures 2D, E**). These findings suggest that the addition of TLR4 to the MVA-based vaccine might affect the immunological context in which the antigens are presented to the T cells within the bLNs.

### TLR9 agonist reduced the number and cumulative size of MVA-induced BALT without affecting the MVA-SARS-2-S-induced recruitment of lymphocytes into the lungs

**Figure 3:**
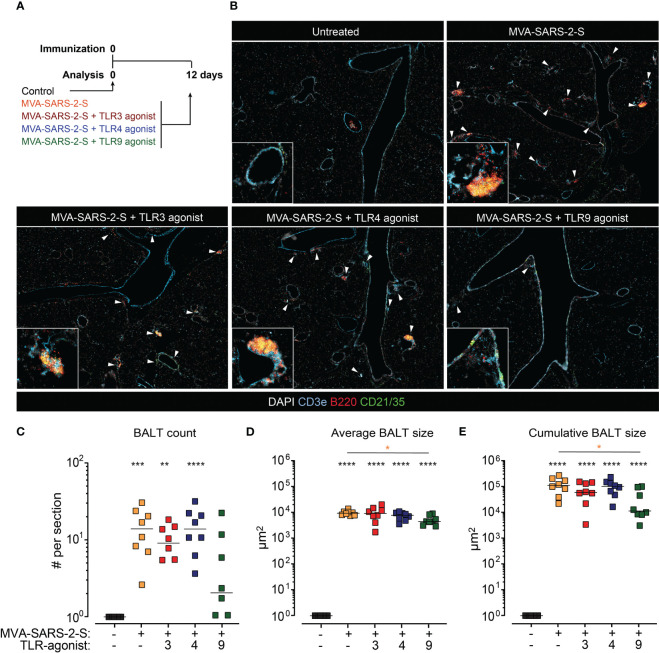
The addition of TLR9 agonist to MVA-SARS-2-S reduced the formation of bronchus associated lymphoid tissue (BALT). **(A)** Scheme of immunization protocol. **(B)** Representative photomicrographs of lung sections reveal induction of BALT (labeled with white arrowheads) 12 days after vaccination. BALT **(C)** count, **(D)** average size, and **(E)** cumulative size calculated as sum of all individual lymphoid structures per lung section. **(C–E)** Pooled data from four independent experiments with n = 7-8 mice per group. Individual values (symbols) and mean group value (lines). Statistical analysis was done on log-transformed using Brown-Forsythe ANOVA test followed by Dunnett’s T3 multiple comparisons test. *p < 0.05, **p < 0.01, ***p < 0.001, ****p < 0.0001. Black stars - difference to control group; Orange stars – difference between groups receiving vaccine and vaccine mixed with TLR agonists, as denoted by the line.

To investigate the effect of TLR agonists on adaptive immune responses induced by respiratory delivery of MVA-SARS-2-S, we next analyzed samples at day 12 p.i. (**Figure 3A**), when both cellular and humoral anti-S responses could be measured (Bošnjak et al., 2021). We initially determined the effect of TLR agonists on the MVA-SARS-2-S induction of BALT, tertiary lymphoid structures composed of T and B cell areas and located in the perivascular space of large blood vessels and next to large airways. Histological analysis of lung sections indicated that TLR3 and TLR4 agonists did not affect MVA-induced BALT formation (**Figure 3**). In contrast, we found significantly reduced numbers of BALT structures with smaller average sizes on lung sections of mice immunized with MVA-SARS-2-S in combination with TLR9 agonists (**Figure 3**). These data suggested that TLR9 agonists could have suppressive rather than enhancing effects on the MVA-SARS-2-S immune response.

**Figure 4:**
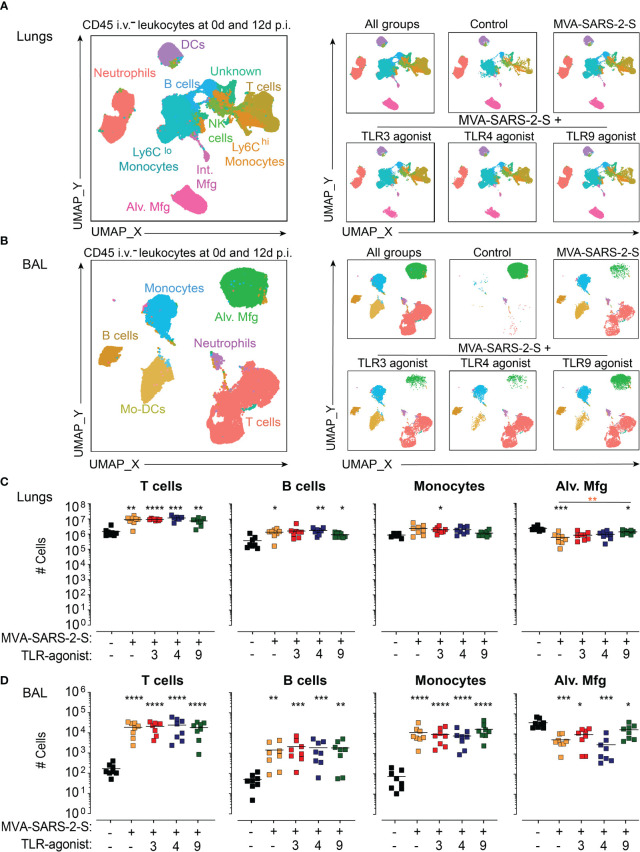
TLR agonists do not affect -induced leukocyte recruitment into the lungs at 12 day after i.n. MVA-SARS-2-S administration. **(A, B)** Spectral flow cytometry analysis of the cellular composition of lung **(A)** and broncho-alveolar lavage (BAL) **(B)** depicted as UMAP plots of concatenated samples of two representative mice from each experimental group. Alv. Mfg – alveolar macrophages; Int. Mfg – interstitial macrophages. **(C, D)** Absolute cell numbers of T cells, B cells, monocytes, and alveolar macrophages in lung **(C)** and BAL **(D)**. Pooled data from four independent experiments with n = 8 mice per group. Individual values (symbols) and mean group value (lines). Statistical analysis was done on log-transformed using Brown-Forsythe ANOVA test followed by Dunnett’s T3 multiple comparisons test. *p < 0.05, **p < 0.01, ***p < 0.001, ****p < 0.0001. Black stars - difference to control group; Orange stars – difference between groups receiving vaccine and vaccine mixed with TLR agonists, as denoted by the line.

In line with our previous observations (Bošnjak et al., 2021), analysis of leukocyte cell subsets in mouse lungs using spectral flow cytometry revealed that i.n. administration of MVA-SAR-2-S induced a significant accumulation of T cells, B cells, interstitial macrophages (Int. mfg), cDC1s, and cDC2s in lungs and BAL (**Figure 4**; **Supplementary Figures 3, 4**). On the other hand, the number of alveolar macrophages (Alv. Mfg), one of the prime cellular targets of MVA (Halle et al., 2009), was still not recovered at this time point after MVA-SARS-2-S administration (**Figures 4D, E**). The TLR agonists did not significantly affect the numbers of infiltrating leukocytes, apart from a 2.2x higher number of the alveolar macrophages in the group vaccinated with TLR9 agonists (**Figure 4**; **Supplementary Figures 3, 4**). Thus, the administered doses of TLR agonists do not lead to the overt adaptive immune response to the MVA-SARS-2-S vaccination that might negatively affect lung function.

### Mice treated with a combination of TLR3 agonists and MVA-SARS-2-S possess increased numbers of S-specific CD8+ T cells in the lungs

**Figure 5:**
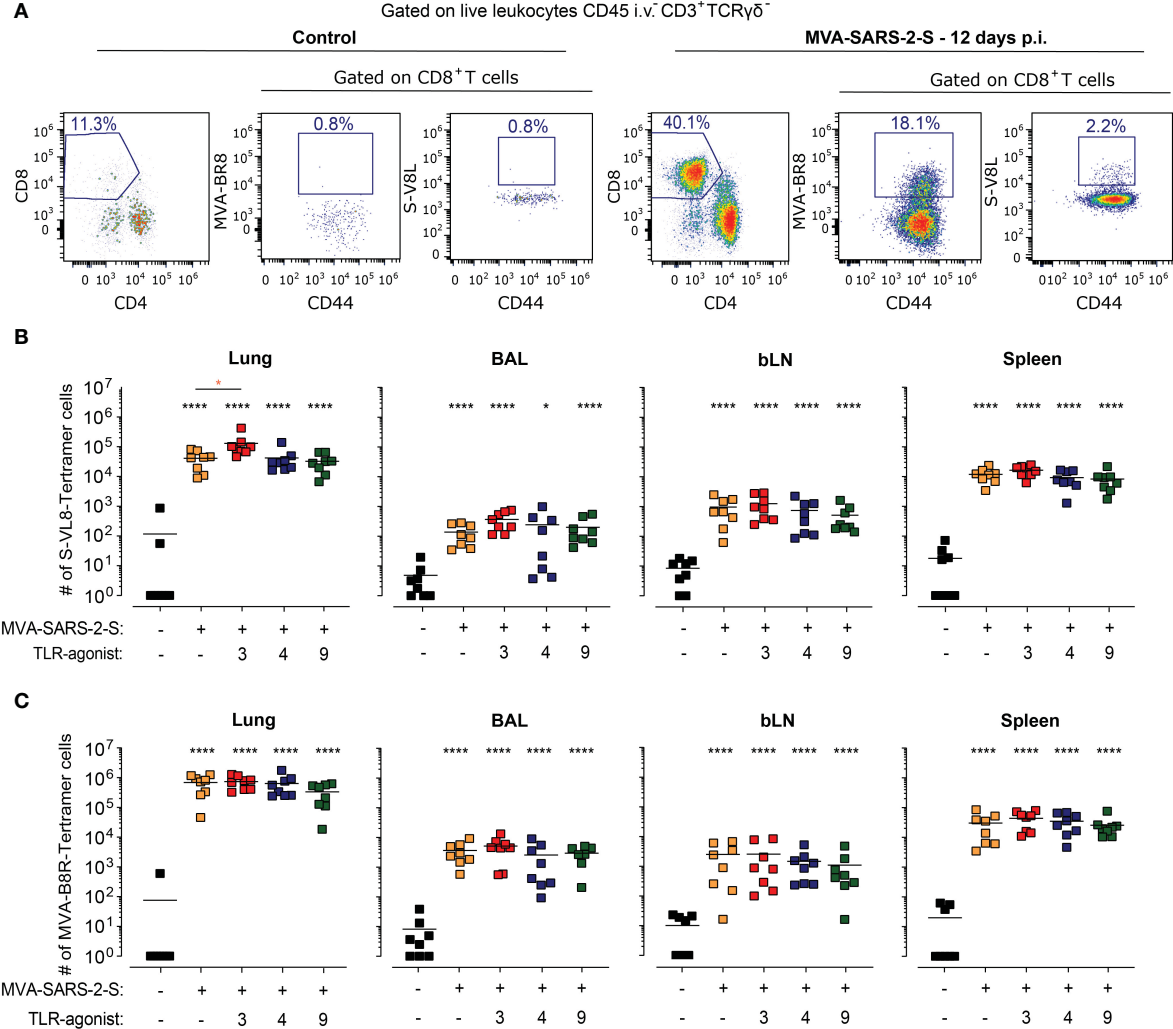
TLR3 agonist (Poly(I:C)) administration enhances the MVA-SARS-2-S-induced cellular response to the spike (S). **(A)** Representative plots showing the percentage of lung S-VL8-tetramer CD8^+^CD44^hi^ T cells in control and MVA-SARS-2-S vaccinated groups. **(B, C)** Absolute cell counts of S-VL8-tetramer^+^CD8^+^CD44^hi^ T cells **(B)** and MVA-B8R-tetramer^+^CD8^+^CD44^hi^ T cells **(C)** in different organs. **(B, C)** Pooled data from four independent experiments with n = 8 mice per group. Individual values (symbols) and mean group value (lines). Statistical analysis was done on log-transformed using Brown-Forsythe ANOVA test followed by Dunnett’s T3 multiple comparisons test. *p < 0.05, ****p < 0.0001. Black stars - difference to control group; Orange stars – difference between groups receiving vaccine and vaccine mixed with TLR agonists, as denoted by the line.

Irrespectively of the addition of TLR agonists, at day 12 p.i. MVA-SARS-2-S immunization increased the number of both CD8^+^ and CD4^+^ T cells not only within lungs and BAL but also in bLNs and spleen (**Supplementary Figures 5A–C**), confirming the existence of local and systemic T cell immune responses to the vaccine. To determine whether the effect of TLR agonists influenced the antigen-specific CD8^+^ immune response, we stained the samples using S-V8L and MVA-B8R tetramers to determine the number of CD8^+^ T cells specific for S and MVA immunodominant peptides, respectively (Bošnjak et al., 2021) (**Figure 5A**). In line with our previous results (Bošnjak et al., 2021), immunization with a single i.n. application of MVA-SARS-2-S induced local and systemic S-specific cellular immune response, characterized by accumulation of S-specific CD8^+^ T cells in lungs, BALs, bLNs, and spleens. Importantly, already at day 12 p.i. lung S-specific CD8^+^ T cells showed high expression of CD103 and CD69 (**Supplementary Figures 5D, E**), immune molecules that mark tissue-resident memory T cells in the lungs.

Importantly, we found that mice immunized with MVA-SARS-2-S in combination with TLR3 agonist had 3.2x higher numbers of S-specific CD8^+^ T cells in the lungs compared to the mice that received the vaccine alone (**Figure 5B**). This increase was also noticeable in BAL (2.7x more cells, not statistically significant), but not in bLNs or spleen (**Figure 5B**), suggesting that the addition of TLR3 agonist affected the local accumulation of S-specific CD8^+^ T cells. In contrast, the TLR3 agonist did not affect the number of MVA-specific CD8^+^ T cells (**Figure 5C**). Similarly, the addition of TLR4 or TLR9 agonists did not affect the S-specific or MVA-specific CD8^+^ T cell number in any of the analyzed organs and tetramers used (**Figures 5B, C**). Taken together, our data suggest that TLR3 agonist has the potential to boost antigen-specific immune responses in the lungs after respiratory immunization with MVA-based vaccines.

### The co-administration of TLR agonists had no boosting effects on humoral S-specific immune responses

**Figure 6:**
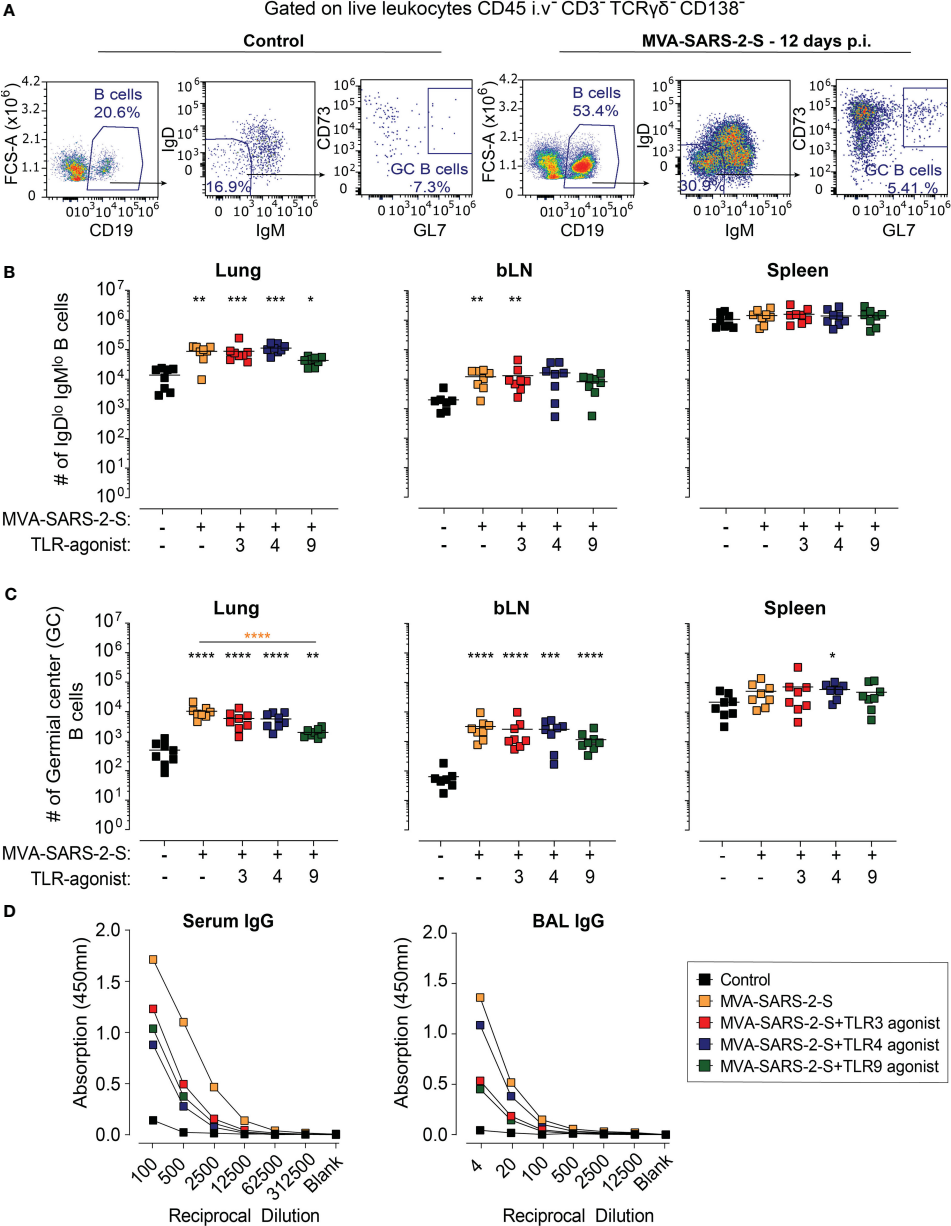
The addition of TLR agonists to MVA-SARS-2-S does not boost humoral response following intranasal vaccination. **(A)** Representative plots showing percentages of total, isotype-switched (IgD^lo^ IgM^lo^), and germinal center **(GC)** B cells in the lungs of indicated groups. **(B, C)** Absolute cell counts of IgD^lo^ IgM^lo^ B cells **(B)** and GC B cells **(C)** in lungs, bronchial lymph nodes (bLNs), and spleens. **(D)** Spike-specific antibodies in serum and broncho-alveolar lavage fluid (BAL) were quantified using ELISA and presented as mean group OD value. **(B–D)** Data from four separate experiments were combined, with a total of 8 mice per group. **(B, C)** Each data point is represented by a symbol, and the mean value for each group is shown with a line. Statistical analysis was performed using Brown-Forsythe ANOVA test, followed by Dunnett’s T3 multiple comparisons test, on logarithmically transformed values. Significance levels are denoted by asterisks: *p < 0.05, **p < 0.01, ***p < 0.001, ****p < 0.0001. Black stars - difference to control group; Orange stars – difference between groups receiving vaccine and vaccine mixed with TLR agonists, as denoted by the line.

Next, we also examined the effect of TLR agonists on the humoral response-induced vaccination. Using spectral flow cytometry, we initially determined the effect of TLR agonists on the number of isotype-switched (CD19^+^IgD^lo^IgM^lo^) and germinal center (GC; CD19^+^IgD^lo^IgM^lo^CD73^+^GL7^+^) B cells. Single i.n. MVA-SARS-2-S administration increased the number of isotype-switched and GC B cells not only in draining bLNs but also within the lungs, which is in line with the formation of BALT in the immunized animals (**Figures 6A–C**). The GC B cell number also increased in the spleen, albeit not significantly (**Figure 6C**). Surprisingly, TLR agonists had rather opposing than stimulating effects on the numbers of isotype-switched and GC B cells, especially TLR9 which in addition suppressed the GC B cell number for on average 81% and 64% in the lungs and bLNs, respectively. The GC B cell numbers within the lungs appeared to be reduced, albeit not significantly, in the groups that received the vaccine mixed with TLR3 or TLR4 agonists (**Figure 6C**).

Finally, we examined the effect on TLR adjuvants of the levels of anti-S antibodies in serum and BAL. Consistent with our previous results (Bošnjak et al., 2021), the MVA-SARS-2-S-treated group displayed relatively low levels of anti-S immunoglobulin (Ig) G antibodies in serum and BAL (**Figure 6D**). Surprisingly, co-administration of the vaccine with either TLR3, TLR4, or TLR9 agonist reduced the titer of anti-S IgG in both serum and BAL (**Figure 6D**). Of note, no anti-S IgA antibodies could be detected in either serum or BAL of MVA-SARS-2-S-immunized mice, irrespectively whether it was administered alone or in combination with TLR adjuvant (data not shown). Collectively, these results demonstrate that the examined TLR agonists have no boosting effects on the humoral immune responses induced by single intranasal vaccination with MVA-SARS-2-S.

## Discussion

**Figure 7:**
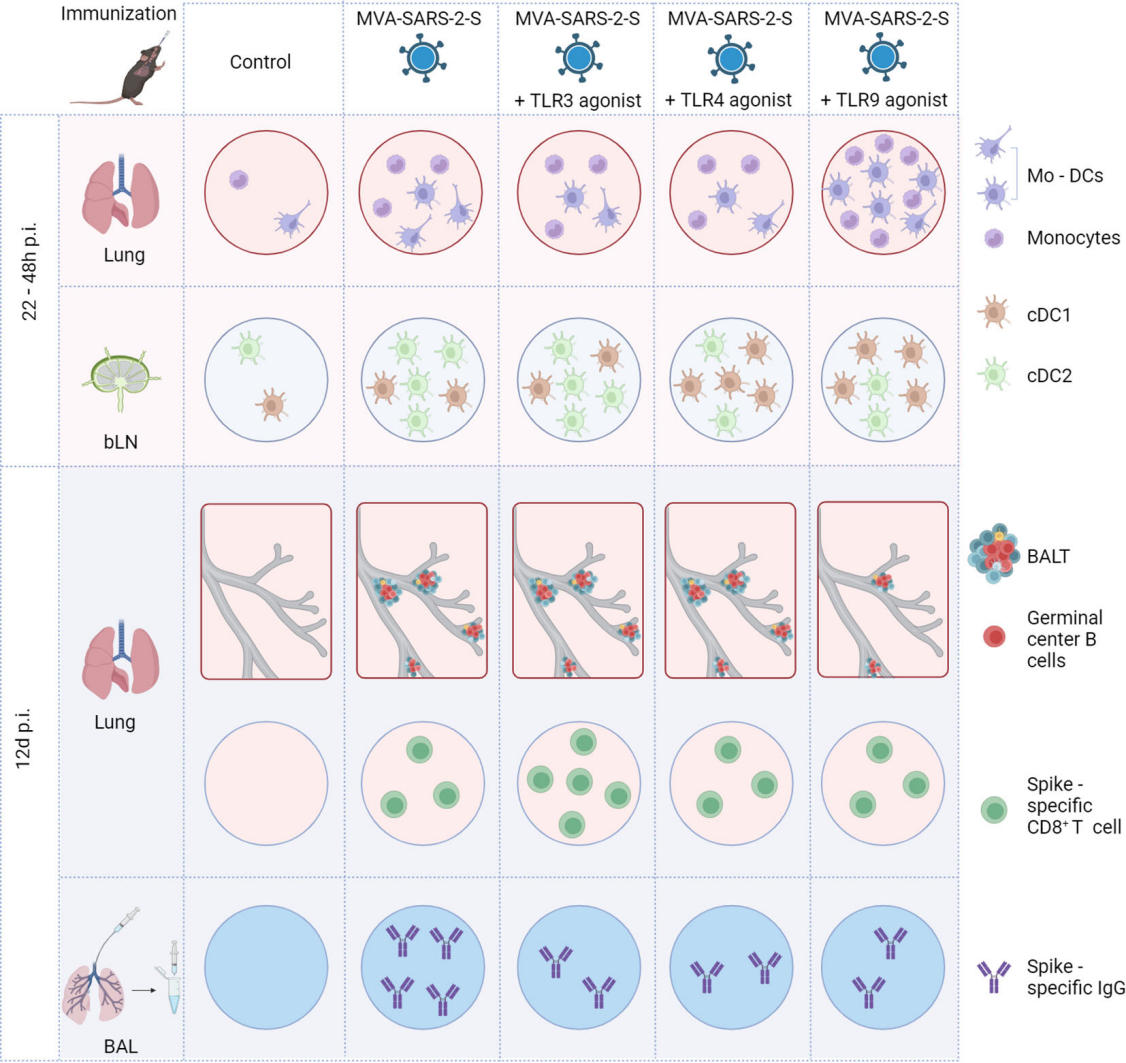
Schematic representation of main effects of TLR agonists on the immune response induced by intranasal immunization with MVA-SARS-2-S. BAL, bronchoalveolar lavage; BALT, bronchus associated lymphoid tissue; bLN, bronchial lymph node; cDC1, conventional dendritic cell type 1; cDC2, conventional dendritic cell type 2; Mo-DCs, monocyte-derived dendritic cells.

The results of this study highlight the complexity of TLR agonists’ adjuvant effects on the immunogenicity of viral vector vaccines, summarized in **Figure 7**. On one hand, the TLR3 agonist, poly(I:C), boosted cellular responses to the respiratory-delivered MVA-based vaccine. On the other hand, none of the investigated TLR agonists boosted S-specific humoral immunity. Additionally, the TLR9 agonist, CpG ODN, had suppressive effects on MVA-induced BALT formation.

The adjuvant effect of TLR3 agonist on the generation of S-specific CD8^+^ T cells within the lungs is in agreement with previous reports indicating that TLR3 agonists can improve the efficacy of live smallpox vaccines (Israely et al., 2014), inactivated influenza vaccines (Ichinohe et al., 2007; Renu et al., 2020), and live influenza vaccine (Overton et al., 2014). Altogether, these data support further investigation of TLR3 agonists as potent adjuvants for respiratory-delivered MVA-based vaccines.

In contrast, the TLR4 and TLR9 agonists did not significantly affect the S-specific CD8^+^ T cell numbers. This was surprising for a TLR9 agonist, as CpG ODNs had adjuvant effects on viral vector vaccines administered intranasally (Bhat et al., 2009; Uppada et al., 2011), intravenously (Fend et al., 2014), subcutaneously (Israely et al., 2014), or intramuscularly (Bhat et al., 2009; Johnson et al., 2009) to mice. Moreover, CpG ODNs showed adjuvant effects on the antigen-specific CD8^+^ T cell responses to intranasally administered MVA (Belyakov et al., 2006). Importantly, Belyakov et al. used a combination of class B and class A CpG ODNs (Belyakov et al., 2006), potent activators of B cells and plasmacytoid DCs (pDCs), respectively. Here, we used only class B CpG ODNs, which induce weaker priming of antigen-specific CD8^+^ T cells *in vitro* than other adjuvants (Papagno et al., 2020). Thus, class B CpG ODNs might need to be administered in a dose higher than 5 µg (which we tested) to exert adjuvant activity on the cellular immune response induced by MVA vaccine. Alternatively, future studies could evaluate if class A CpG ODNs or a mixture of class A and B CpG ODNs have more vigorous adjuvant effects on intranasally delivered MVA vaccines than class B CpG ODNs.

TLR4 agonists also did not stimulate the S-specific CD8^+^ T cell response induced by intranasal administration of MVA-SARS-2-S. Others reported that TLR4 agonists boosted cellular adaptive responses to adenoviral-based vector vaccines after intranasal (Li et al., 2016) and intramuscular (Rhee et al., 2010) administration in mice. Interestingly, in the latter study, TLR9 agonists had no effect, whereas TLR3 agonist even significantly suppressed adenovirus-induced cellular immune responses (Rhee et al., 2010). The opposing effects of TLR3 and TLR4 agonists on adenoviral vector immunization seem to be caused by their effect on DCs (Lebedeva et al., 2018). In DCs infected with the adenovirus vaccine, TLR4 agonist increased the production of target antigen mRNA and expression of costimulatory molecules CD80, CD86, and CD40. On the other hand, TLR3 agonists inhibited the production of target antigen mRNA and increased only CD40 expression on the surface of DC (Lebedeva et al., 2018). As adenovirus- and MVA-based vaccines induce distinct transcription responses in DC (Sheerin et al., 2021), it is likely that TLR agonists might have distinct effects on the MVA-activated than on adenovirus-activated DCs. In line with this hypothesis are findings that TLR agonists’ immunomodulatory effects can also induce cross-protection against pathogens [reviewed in (Owen et al., 2021)]. For example, co-administration of TLR3 agonists with SARS-CoV-2 protects mice from infection (Tamir et al., 2022). Together, these data highlight the need to carefully select an appropriate TLR agonist whose adjuvant properties would complement and not counteract viral vector vaccine-induced effects on DCs.

Of note, DCs are a very heterogeneous group of cells with divergent antigen presentation and migration properties [reviewed in (Worbs et al., 2017; Kawasaki et al., 2022)]. Within the lungs, DCs are broadly divided into CD103^+^ cDC1s, CD11b^+^ cDC2s, and pDCs (Kawasaki et al., 2022). Importantly, in humans, cDC1s express TLR3, TLR9, and TLR10, and cDC2s express TLR2, TLR4, TLR5, TLR6, and TLR8 (Collin and Bigley, 2018), thus making them differentially sensitive to activation with different TLR agonists. Additionally, cDC1s appear to be main inducers of CD8^+^ T cell responses, while cDC2s are more specialized for induction of CD4^+^ T cell responses (Kawasaki et al., 2022). We found that the TLR4 agonist increased the proportion of cDC2 cells in bLNs after MVA-SARS-2-S vaccination. Although we did not formally address the question of whether those cDCs carried the MVA-encoded antigens, this finding still suggests that TLR4 agonist could stimulate vaccine-induced helper T cell response and, consequently, production of S-specific antibodies. However, none of tested TLR agonists boosted serum or BAL anti-S antibody levels induced by MVA-SARS-2-S. This effect was unexpected, as numerous examples from literature show that TLR adjuvants are potent stimulators of humoral immunity [reviewed in (Duthie et al., 2011; Kaur et al., 2022)]. Moreover, intranasally administered peptide RS09, a TLR4 agonist, expressed in the adenoviral vaccine (Li et al., 2016) and TLR5 adjuvant flagellin expressed in the MVA vaccine (Sanos et al., 2018) and vesicular stomatitis virus (Ahmed et al., 2010) potentiated vaccine-induced humoral responses. Those results suggest that appropriate TLR adjuvant should be properly formulated to stimulate vaccine-induced humoral immunity. It is now well accepted that the adjuvant effects of TLR agonists can be improved by their conjugation with the antigen, high-density presentation, and/or by slowing down the rate of their release [reviewed in (Kaur et al., 2022)]. Therefore, suitable TLR adjuvants, such as TLR5 adjuvant flagellin or TLR9 adjuvant CpG, could be incorporated into the genome of the novel MVA-based COVID-19 vaccine candidates. Alternatively, more complex formulations of TLR agonists suitable for respiratory delivery might provide better co-presentation. Another possibility could be to combine different adjuvants, as combinations of TLR3 or TLR4 agonists with TLR9 agonist lead to enhanced T helper type 1-polarized immune response (Napolitani et al., 2005).

Respiratory delivery of MVA to mice leads also to the development of BALT, a tertiary lymphoid structure in which B and T cells are organized like in secondary lymphoid organs and can serve as a priming site for T cells (Halle et al., 2009). Interestingly, we now found that the addition of TLR9-agonist to the MVA inhibited BALT formation. Although block of DC emigration from the lungs is crucial for BALT induction (Fleige et al., 2018), the number and type of activated DCs in the bLNs of mice that received MVA-SARS-2-S in combination with TLR9 agonist were not significantly different when compared to the group vaccinated with MVA-SARS-2-S. Therefore, it seems that TLR9 agonists affected the BALT formation and/or maintenance using mechanisms other than directly affecting DC migration, which will be addressed in future studies.

Altogether, our data indicate the capacity of TLR agonists to modulate vaccine-induced mucosal immune responses. The most promising results were achieved with the TLR3 agonist, warranting further research on the dose-sparing effects of the TLR3 agonist’s optimized formulations on the MVA-SARS-2-S-induced cellular response. On the other hand, other TLR agonists, especially flagellin targeting TLR5, could boost the humoral immune response induced by MVA-SARS-2-S. Further optimization adjuvants targeting TLRs could pave the way for potent single-dose MVA-based vaccine formulations against respiratory pathogens.

### Limitations of the study

In this study we have not addressed the effect of TLR agonists on helper T cell response, or whether selected TLR adjuvants provide dose-sparing effects for the MVA vaccines. Those studies should also use MVA vaccine with stabilized S protein, which was recently shown to induce comparable cellular but superior humoral responses than MVA-SARS-2-S (Pérez et al., 2021; Meyer zu Natrup et al., 2022; Kalodimou et al., 2023).

## Data availability statement

The original contributions presented in the study are included in the article/**Supplementary Material**. Further inquiries can be directed to the corresponding authors.

## Ethics statement

All animal procedures were conducted following the European and national regulations for animal experimentation, including the European Directive 2010/63/EU and the Animal Welfare Acts in Germany, and were approved by the Lower Saxony State Office for Consumer Protection and Food Safety (LAVES; TVV 17/2586).

## Author contributions

KD: Data curation, Investigation, Methodology, Writing – original draft. SW: Investigation, Methodology, Writing – review & editing. JR: Investigation, Methodology, Writing – review & editing. AJ: Investigation, Methodology, Writing – review & editing. AV: Funding acquisition, Methodology, Writing – review & editing. GS: Funding acquisition, Methodology, Writing – review & editing. RF: Conceptualization, Supervision, Writing – review & editing. BB: Conceptualization, Data curation, Methodology, Supervision, Writing – original draft.
