# Supplementary material for: The effect of Toll-like receptor agonists on the immunogenicity of MVA-SARS-2-S vaccine after intranasal administration in mice
Source: Front Cell Infect Microbiol. 2023 Oct 3;13:1259822. doi: 10.3389/fcimb.2023.1259822 (PMC10580083; doi:10.3389/fcimb.2023.1259822)
Supplement: Supplementary file 1 [file DataSheet_1.docx]

***Supplementary Figures and Tables***

The effect of Toll-like receptor agonists on the immunogenicity of MVA-SARS-2-S vaccine after intranasal administration in mice

Kim Thi Hoang Do^1^, Stefanie Willenzon^1^, Jasmin Ristenpart^1^, Anika Janssen^1^, Asisa Volz^2,3^, Gerd Sutter^3,4^, Reinhold Förster^1,5,6,†,*^, Berislav Bošnjak^1,5,†,*^

^1^ Institute of Immunology, Hannover Medical School, 30625 Hannover, Germany

^2^ Institute for Virology, University of Veterinary Medicine Hannover, 30559 Hannover, Germany

^3^ German Centre for Infection Research (DZIF), Partner site 80539 Munich, Germany

^4^ Division of Virology, Department of Veterinary Sciences, LMU Munich, 80539 Munich, Germany

^5^ Cluster of Excellence RESIST (EXC 2155), Hannover Medical School, 30625 Hannover, Germany

^6^ German Centre for Infection Research (DZIF), Partner site 30625 Hannover, Germany

† These authors contributed equally to this work

*** Correspondence:**Dr. Berislav Bošnjak, Institute of Immunology, Hannover Medical School, Carl-Neuberg Straße 1, 30625 Hannover, Germany, phone: +49 511 532 9729, email: bosnjak.berislav@mh-hannover.de

Prof. Dr. Reinhold Förster, Institute of Immunology, Hannover Medical School, Carl-Neuberg Straße 1, 30625 Hannover, Germany, phone: +49 511 532 9721, email: foerster.reinhold@mh-hannover.de

**Supplementary Figures**


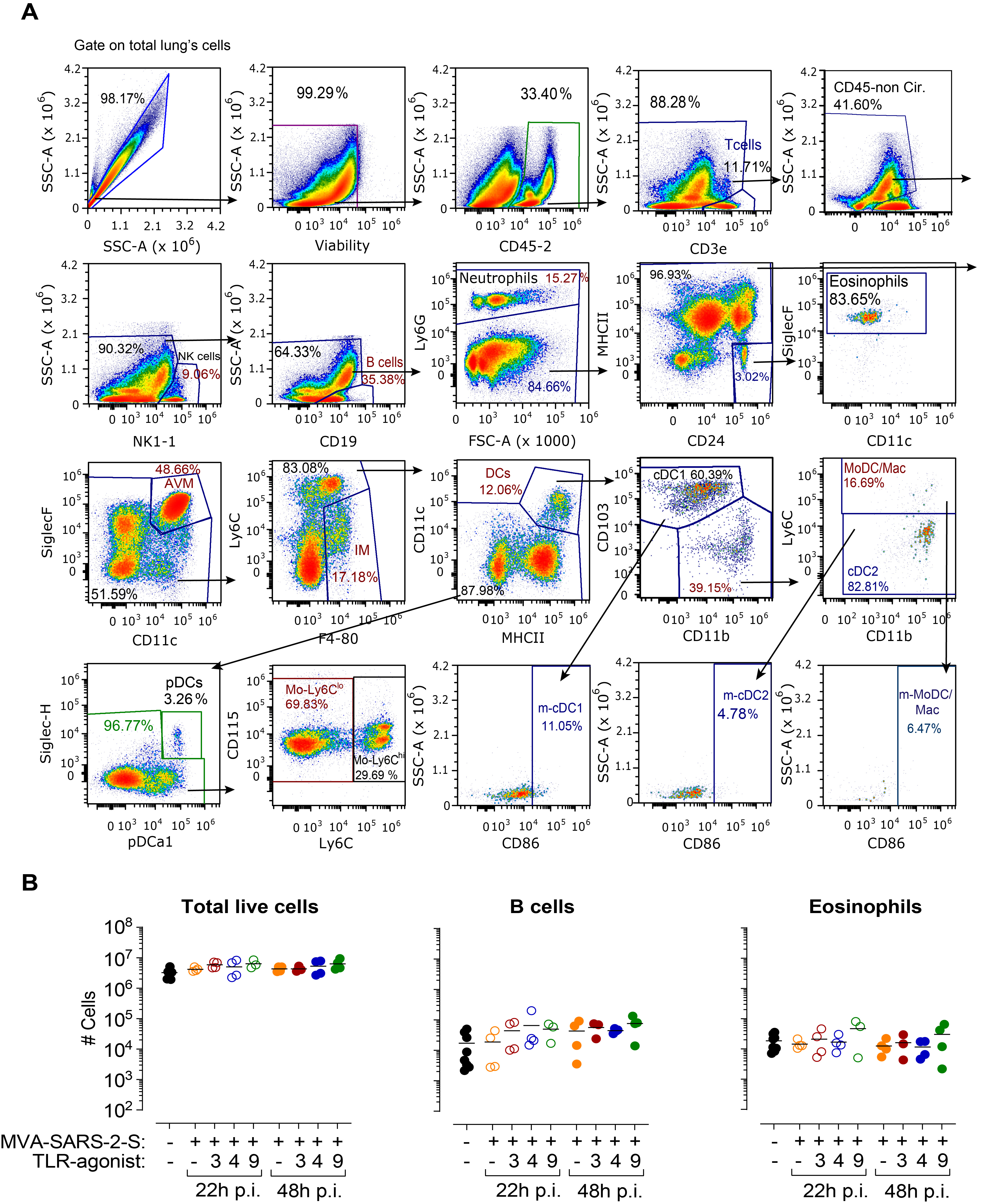


Supplementary Figure 1: Gating strategy and quantification of leukocytes recruited to the lung after intranasal application of MVA-SARS-2-S alone or in combination with indicated TLR adjuvants. At day 0, C57BL/6 Mice were intranasally immunized with 10^7^ PFU of MVA-SARS-2-S alone or in combination with 5 µg LPS from *Escherichia coli* O55:B5, 5 µg CpG ODN 1826, or 3 µg poly(I: C). Lungs were collected and analyzed at 22 and 48 hours (h) (Immunization protocol scheme in Fig. 1A). (A) Pseudocolor plots depicting the gating strategy for indicated cell populations in representative mouse lungs at 22 hours post-infection MVA-SARS-2-S infected mouse at 2, analyzed with panel #1 (Supplementary Table S1). (B) Absolute cell counts of total isolated live cells, B cells, and eosinophils in the lungs at 22 and 48 h post-infection. Pooled data from 2 independent experiments with n = 4 per group. Shown are individual values (symbols) and mean group values (line). Statistical analysis was done on log-transformed values using the Brown-Forsythe ANOVA test followed by Dunnett’s T3 multiple comparisons test.


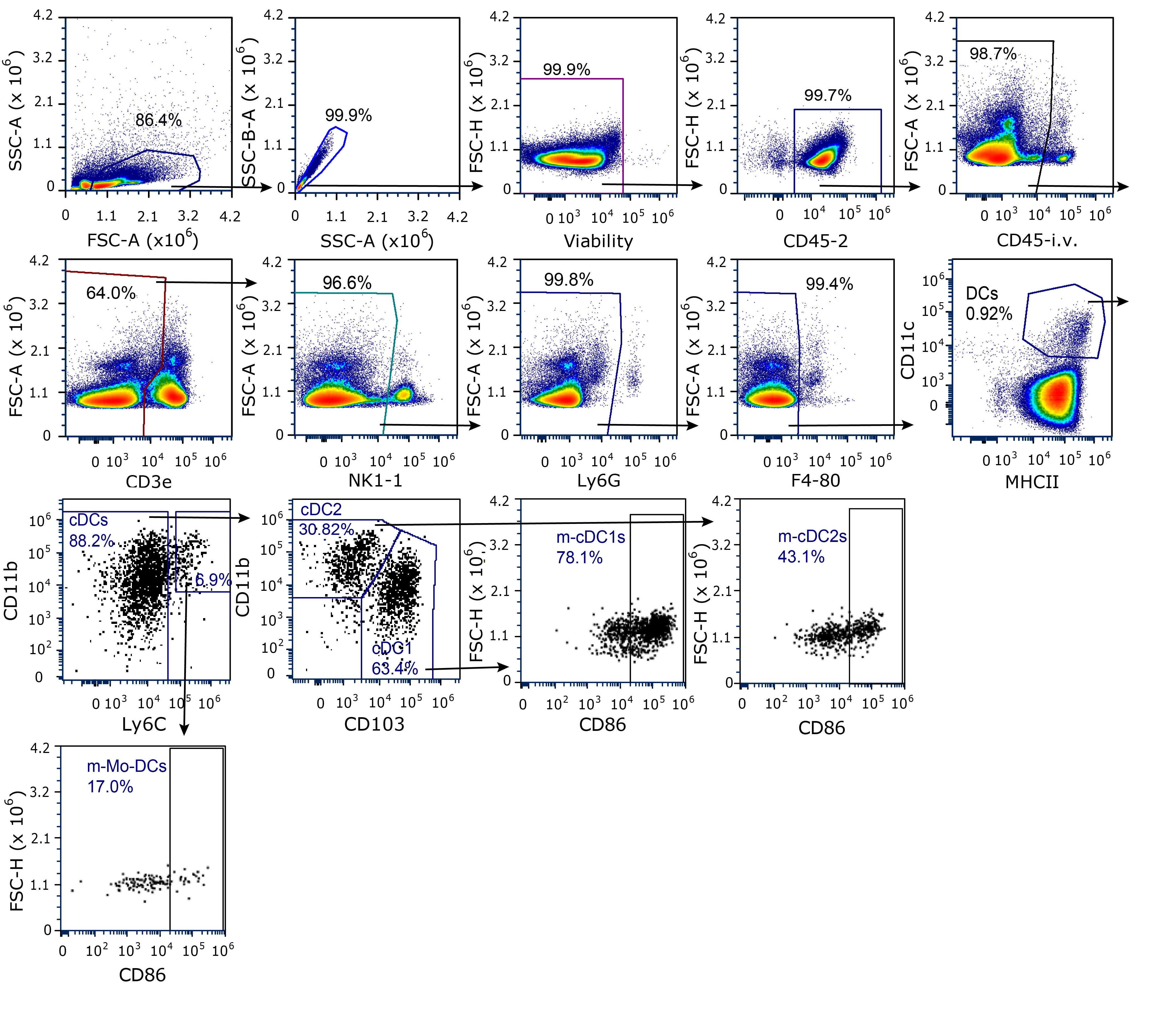


Supplementary Figure 2: Gating strategy for dendritic cell (DCs) subpopulations in bronchial lymph nodes. Pseudocolor plots illustrating the gating strategy for indicated cell populations in representative mouse bronchial lymph nodes (bLNs) at 22 hours post-infection MVA-SARS-2-S analyzed with panel #2 (Supplementary Table S1).


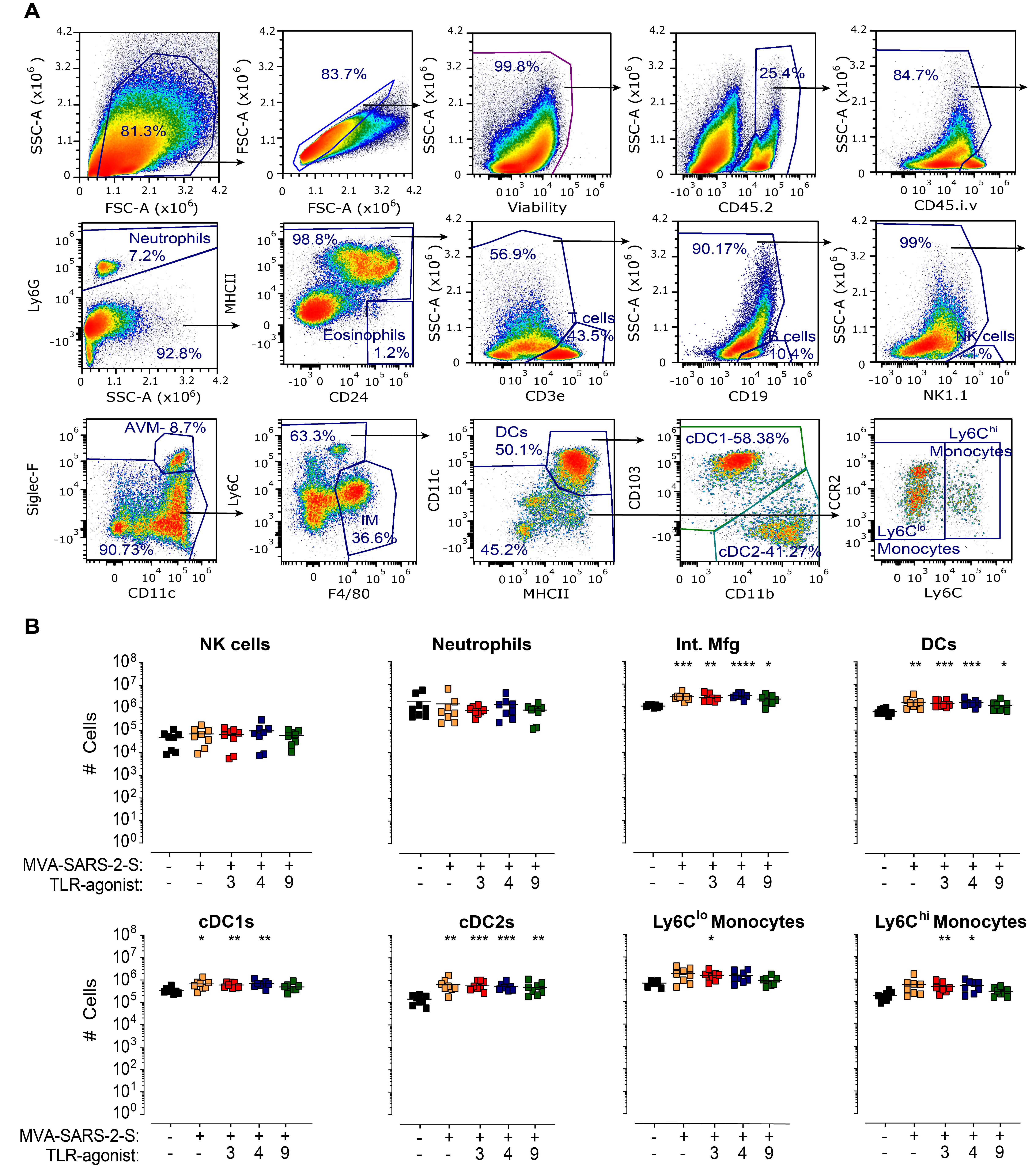


Supplementary Figure 3: Gating strategy and quantification of leukocytes recruited to the lung at day 12 after intranasal application of MVA-SARS-2-S alone or in combination with indicated TLR adjuvants. C57BL/6 Mice were intranasally immunized at day 0 with 10^7^ PFU of MVA-SARS-2-S alone or in combination with TLR agonists and analyzed at day 12 (Immunization protocol scheme is depicted in Fig. 3A). (A) Pseudocolor plots displaying gating strategy of indicated cell populations in representative lungs of a mouse infected with MVA-SARS-2-S and analyzed with staining panel #3 (Supplementary Table S1). (B) Absolute cell counts of lung NK cells, neutrophils, interstitial macrophages (Int. Mfg), conventional dendritic cells type 1 and type 2 (cDC1 and cDC2, respectively), Ly6C^lo^ monocytes, and Ly6C^hi^ monocytes at 12 days p.i. Pooled data from four independent experiments with a total of 8 mice per group. Data are displayed as individual values (symbols) and mean group values (lines). Statistical analysis was done on log-transformed values using ordinary or Brown-Forsythe ANOVA test followed by Dunnett’s T3 multiple comparisons test. *p < 0.05, **p < 0.01, ***p < 0.001, ****p < 0.0001. The black stars represent differences between the treatment groups and the control group.


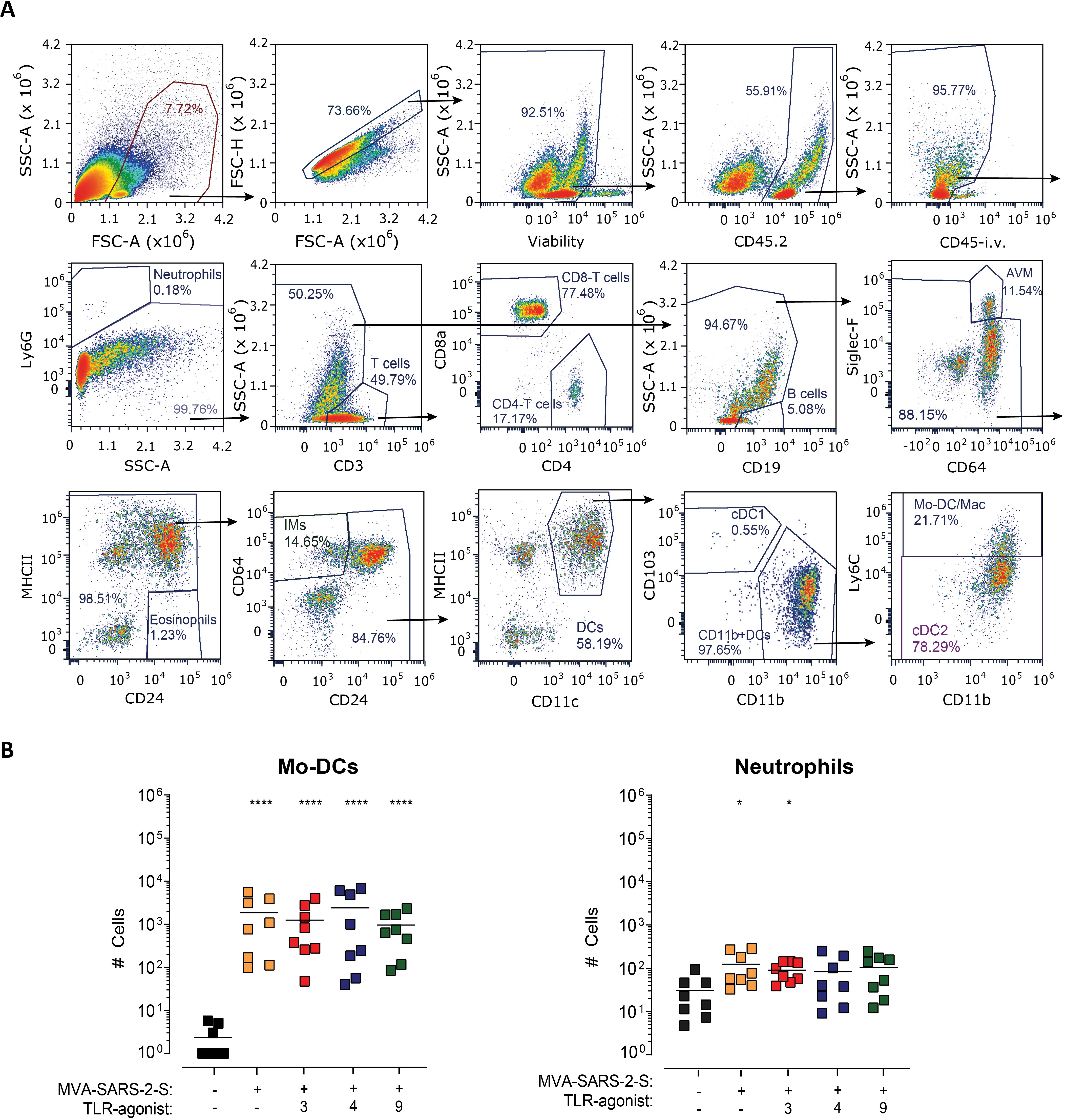


Supplementary Figure 4: Gating strategy and quantification of leukocytes recruited to the bronchoalveolar lavage (BAL) at day 12 after intranasal application of MVA-SARS-2-S alone or in combination with indicated TLR adjuvants. (A) Pseudocolor plots displaying the gating strategy of indicated cell populations in representative BAL of a mouse infected with MVA-SARS-2-S and analyzed with staining panel #4 (Supplementary Table S1). (B) Absolute cell counts of monocyte-derived dendritic cells (Mo-DCs) and neutrophils present in BAL at 12 days p.i. Pooled data from four independent experiments with a total of 8 mice per group. Data are displayed as individual values (symbols) and mean group values (lines). Statistical analysis was done on log-transformed values using ordinary or Brown-Forsythe ANOVA test followed by Dunnett’s T3 multiple comparisons test. *p < 0.05, **p < 0.01, ***p < 0.001, ****p < 0.0001. The black stars represent differences between the treatment groups and the control group.


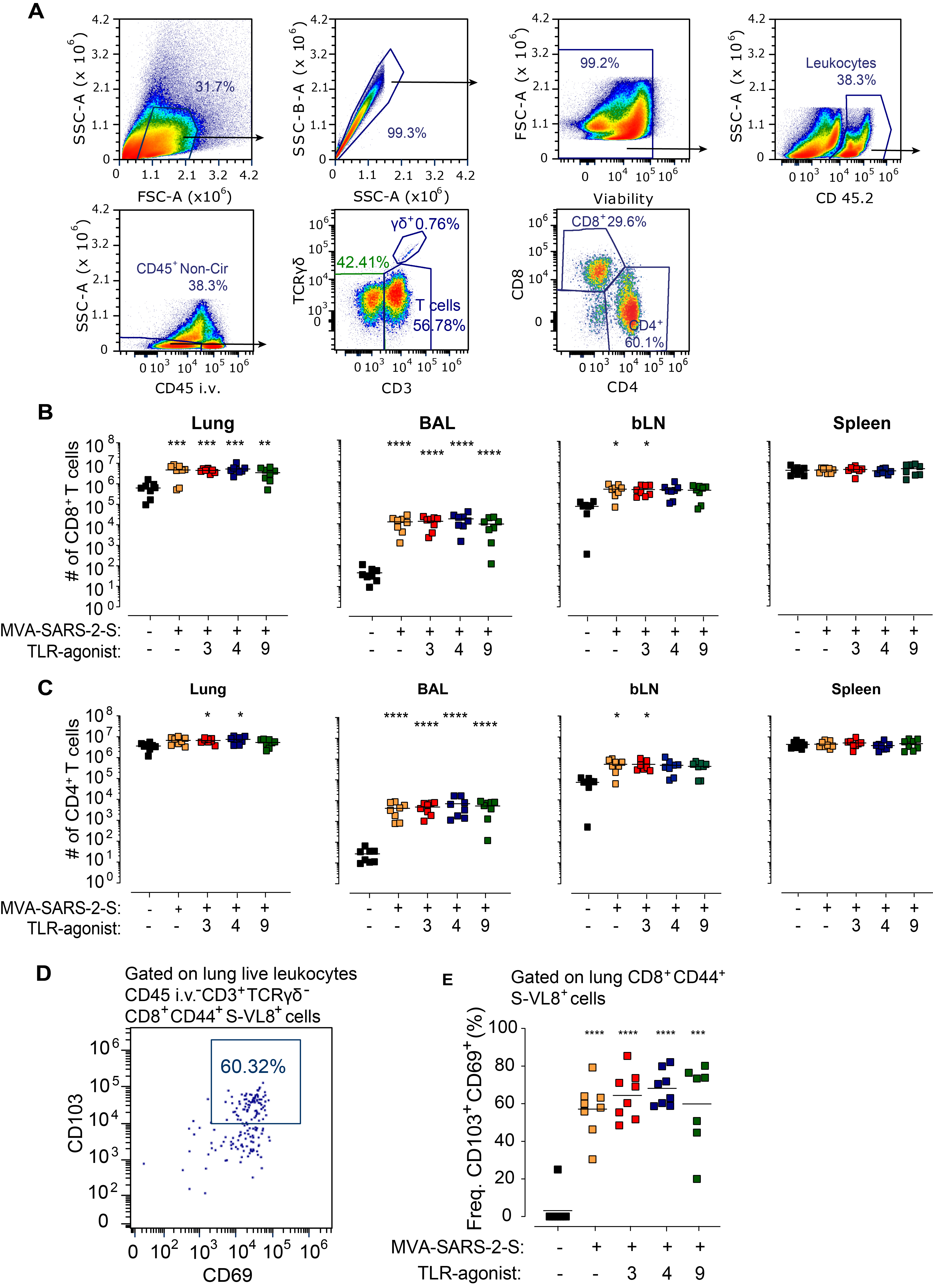


Supplementary Figure 5: Characterization of T cells in different organs after vaccination with a single intranasal (i.n.) dose of MVA-SARS-CoV-2-S with or without TLR agonists. (A) The gating strategy used to identify T cell populations in the lung was based on the staining panel #5 described in Supplementary Table S1. Pseudo-color plots show data from a representative mouse analyzed 12 days after vaccination with MVA-SARS-2-S. (B,C) Absolute cell counts of CD8^+^ (B) and CD4^+^ (C) T cells in the lungs, broncho-alveolar lavage (BAL), bronchial lymph nodes (bLN), and spleen. (D) Representative dot plot of Spike-specific CD8^+^ T cells (CD8^+^CD44^+^S-VL8^+^ T cells). (E) Frequency of CD103^+^CD69^+^ T cells within resident spike-specific CD8^+^ T cells in the mouse lungs. (B,C,E) The data were pooled from four independent experiments with a total of 8 mice per group. Individual values are represented by symbols, and mean group values are indicated by a line. Statistical analysis was performed on log-transformed values using the Brown-Forsythe ANOVA test followed by Dunnett’s T3 multiple comparisons test. Significance levels are denoted as follows: *p < 0.05, **p < 0.01, ***p < 0.001, ****p < 0.0001. Black stars - difference to control group.


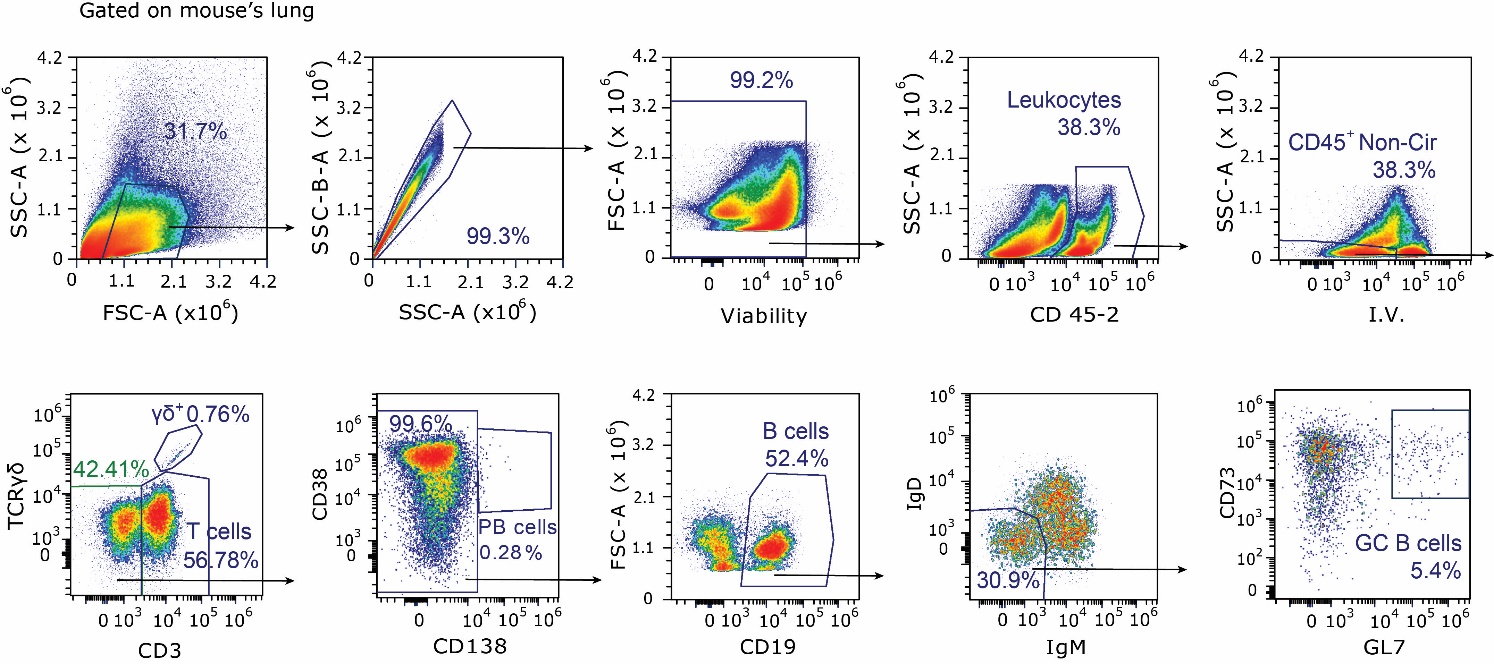


Supplementary Figure 6: Gating strategy for total, isotype-switched (IgD^lo^ IgM^lo^), and germinal center (CD73^+^GL7^+^) B cell populations. Pseudocolor plots illustrating the gating strategy for indicated cell populations in representative mouse lungs at 12 days post-infection MVA-SARS-2-S analyzed with panel #5 (Supplementary Table S1).

**Supplementary Tables**

**Supplementary table S1. List of antibodies used.** Panel 1 – Staining of lung cells at 22 and 48 hours post infection, Panel 2 – staining of bronchial lymph node cells at 22 and 48 hours post infection; Panel 3 – staining of lung cells at 12 days post infection; Panel 4 – staining of bronchoalveolar lavage cells at 12 days post infection; Panel 5 – staining of lung, spleen, and bronchoalveolar lavage cells at 12 days post infection; NA – not applicable.

| **Antibody** | **Clone** | **Source** | **Identifier** | **Panel** |
| --- | --- | --- | --- | --- |
| *Analysis of cells isolated from lungs, broncho-alveolar lavage, bronchial lymph node and spleen* | | | | |
| BUV661 anti-mouse Ly6G | 1A8 | BD | Cat# 741587; RRID: AB_2871000 | 1,2,3 |
| eF450 anti-mouse CD11b | M1/70 | Thermo Fisher Scientific | Cat# 48-0112-82; RRID: AB_1582236 | 1,2,3 |
| PE-Texas Red anti-mouse CD11b | M1/70.15 | Thermo Fisher Scientific | Cat# RM2817; RRID: AB_10373548 | 4 |
| BV421 anti-mouse CD103 | 2E7 | BioLegend | Cat# 121422;  RRID: AB_2562901 | 1,2,3 |
| BV785 anti-mouse CD103 | 2E7 | BioLegend | Cat# 121439;  RRID: AB_2800588 | 4 |
| BV711 anti-mouse CD103 | 2E7 | BioLegend | Cat# 121435; RRID: AB_2686970 | 5 |
| BV650 anti-mouse CD11c | N418 | BioLegend | Cat# 117339; RRID: AB_2562414 | 1,2,3,4 |
| BV650 anti-mouse CD44 | IM7 | BioLegend | Cat# 103049; RRID: AB_2562600 | 5 |
| BV605 anti-mouse F4/80 | BMB | BioLegend | Cat# 123133; RRID: AB_2562305 | 1,2,3 |
| Spark blue anti-mouse CD19 | 6D5 | BioLegend | Cat# 115566; RRID: AB_2832389 | 1,3,4 |
| BUV661 anti-mouse CD19 | 1D3 | BD | Cat# 612971; RRID: AB_2870243 | 5 |
| Pacific Blue anti-mouse GL7 | GL7 | BioLegend | Cat# 144614; RRID: AB_2563292 | 5 |
| BV510 anti-mouse CD4 | RM4-5 | BioLegend | Cat# 100559; RRID: AB_2562608 | 5 |
| BUV395 anti-mouse CD4 | GK1.5 | BD | Cat# 563790; RRID: AB_2738426 | 4 |
| BV785 anti-mouse CD3e | 145-2C11 | BioLegend | Cat#100355; RRID: AB_2565969 | 1,2,3 |
| AF532 anti-mouse CD3 | 17A2 | Invitrogen | Cat#58-0032-82; RRID: AB_11217479 | 5 |
| PE-Cy5.5 anti-mouse CD3 | 35-0031-82 | Invitrogen | Cat#35-0031-82; RRID: AB_11219266 | 4 |
| BV785 anti-mouse CD138 | 281-2 | BD | Cat#740880; RRID: AB_11209249 | 5 |
| FITC anti-mouse CD45 | 30-F11 | BioLegend | Cat# 109841; RRID: AB_312973 | 1,2,3,4,5 |
| PerCP-Cy5.5 anti-mouse CD45.2 | 104 | Thermo Fisher Scientific | Cat# 45-0454-82, RRID: AB_953590 | 1,2,3,4,5 |
| BV711 anti-mouse CD64 (FcγRI) | X54-5/7.1 | BioLegend | Cat# 139311; RRID: AB_2563846 | 4 |
| PE-Cy7 anti-mouse NK1.1 | PK136 | BioLegend | Cat# 108708; RRID: AB_313395 | 1,2,3 |
| PE anti-mouse Siglec-H | 511.3D3 | Miltenyi | Cat# 130-102-261; RRID: AB_2660877 | 1 |
| PE anti-mouse CD73 | TY/11.8 | BioLegend | Cat# 127206; RRID: AB_2154094 | 5 |
| PE-Dazzle 594 anti-mouse CD69 | H1.2F3 | Biolegend | Cat# 104536; RRID: AB_2565583 | 5 |
| BV510 anti-mouse I-A/I-E | M5/114.15.2 | BioLegend | Cat# 107635; RRID: AB_2561397 | 1,2,3 |
| APC anti-mouse I-A/I-E | M5/114.15.2 | BioLegend | Cat# 17-5321-82; RRID: AB_469455 | 4 |
| AF647 anti-mouse CD24 | M1/69 | BioLegend | Cat# 101818; RRID: AB_493484 | 3 |
| PE anti-mouse CD24 | M1/69 | BioLegend | Cat# 101808; RRID: AB_312841 | 4 |
| AF647 anti-mouse TCRγ/δ | GL3 | BioLegend | Cat# 118134; RRID: AB_2566407 | 5 |
| AF®700 anti-mouse pDCa1 (Bst2) antibody | 927 | BioLegend | Cat# 127038, RRID: AB_2832458 | 1 |
| APC-Cy7 anti-mouse Siglec-F antibody | E50-2440 | BD | Cat# 565527; RRID: AB_2732831 | 1,3,4 |
| BUV395 anti-mouse IgM | R6-60.2 | BD | Cat# 564025; RRID: AB_2738550 | 5 |
| BUV805 anti-mouse IgD | 217-170 | BD | Cat# 749299; RRID: AB_2873673 | 5 |
| BV711 anti-mouse Ly6-C | HK1.4 | BioLegend | Cat# 128037; RRID: AB_2562630 | 1,2,3 |
| PE anti-mouse CD86 | GL1 | BioLegend | Cat# 105008; RRID: AB_313151 | 1, 2 |
| BUV 496 anti-mouse CD115 (CSF-1R) | T38-320 | BD | Cat# 74997; RRID: AB_2874201 | 1 |
| PE-Cy7 anti-mouse Ly6-C | HK1.4 | BioLegend | Cat# 128018; RRID: AB_1732093 | 4 |
| PE anti-mouse CD192 (CCR2) | SA203G11 | BioLegend | Cat# 150610; RRID: AB_2616982 | 3 |
| APC-Cy7 anti-mouse CD38 | 90 | BioLegend | Cat# 102728; RRID: AB_2616968 | 5 |
| APC-R700 anti-mouse CD8 | 53-67 | BD | Cat# 564983; RRID: AB_2739032 | 4,5 |
| Zombie NIR™ Fixable Viability | N/A | BioLegend | Cat# 423106 | 1,2,3,4,5 |
| BV 480-Tetramer | N/A | Tetramer-Shop | Mkb-016 | 4,5 |
| BUV 737- Tetramer | N/A | Tetramer-Shop | Mkb-016 | 4,5 |
| *Antibodies used for histology* | | | | |
| PE-Cy7 anti-mouse CD21/35 antibody | 7E9 | BioLegend | Cat# 123420, RRID: AB_1953277 | NA |
| APC anti-mouse CD3e | 500A2 | BioLegend | Cat# 152306, RRID: AB_2632669 | NA |
| PE anti-mouse B220 (CD45R) | RA3-6B2 | BioLegend | Cat# 103208, RRID: AB_312992 | NA |
| DAPI (4',6-Diamidino-2-Phenylindole, Dihydrochloride) |  | Invitrogen | Cat# D1306 | NA |
